# Combined use of immunohistochemical markers of basal and luminal subtypes in urothelial carcinoma of the bladder: Association with clinicopathological features and outcomes

**DOI:** 10.6061/clinics/2021/e2587

**Published:** 2021-04-16

**Authors:** Juliana Naves Ravanini, Aline Kawassaki Assato, Alda Wakamatsu, Venâncio Avancini Ferreira Alves

**Affiliations:** I Departamento de Patologia, Faculdade de Medicina FMUSP, Universidade de Sao Paulo, Sao Paulo, SP, BR.; II CICAP - Hospital Alemao Oswaldo Cruz, Sao Paulo, SP, BR.; III Patologia Hepatica (LIM-14), Hospital das Clinicas HCFMUSP, Faculdade de Medicina, Universidade de Sao Paulo, Sao Paulo, SP, BR.

**Keywords:** Carcinoma, Urinary Bladder, Cystectomy, Histologic Variants, Tissue Microarray, Immunohistochemistry

## Abstract

**OBJECTIVES:**

Whole genome expression profiles allow the stratification of bladder urothelial carcinoma into basal and luminal subtypes which differ in histological patterns and clinical behavior. Morpho-molecular studies have resulted in the discovery of immunohistochemical markers that might enable discrimination between these two major phenotypes of urothelial carcinoma.

**METHODS:**

We used two combinations of immunohistochemical markers, *i.e.*, cytokeratin (CK) 5 with CK20 and CK5 with GATA3, to distinguish subtypes, and investigated their association with clinicopathological features, presence of histological variants, and outcomes. Upon searching for tumor heterogeneity, we compared the findings of primary tumors with their matched lymph node metastases. We collected data from 183 patients who underwent cystectomy for high-grade muscle-invasive urothelial carcinoma, and representative areas from the tumors and from 76 lymph node metastasis were organized in tissue microarrays.

**RESULTS:**

Basal immunohistochemical subtype (CK5 positive and CK20 negative, or CK5 positive and GATA3 negative) was associated with the squamous variant. The luminal immunohistochemical subtype (CK5 negative and CK20 positive, or CK5 negative and GATA3 positive) was associated with micropapillary and plasmacytoid variants. Remarkably, only moderate agreement was found between the immunohistochemical subtypes identified in bladder tumors and their lymph node metastasis. No significant difference in survival was observed when using either combination of the markers.

**CONCLUSION:**

This study demonstrates that these three routinely used immunohistochemical markers could be used to stratify urothelial carcinomas of the bladder into basal and luminal subtypes, which are associated with several differences in clinicopathological features.

## INTRODUCTION

Bladder cancer (BC) is the fourth most common malignancy in men, with 64,280 new cases and 12,260 deaths expected in 2021 in the United States (1). More than 90% of the BCs are urothelial carcinomas, 25% of which have already invaded the muscle at the time of diagnosis. Radical cystectomy with pelvic lymph node dissection (and neoadjuvant chemotherapy when appropriate) is the primary treatment for muscle-invasive BC, with a median overall survival of 48 months (2,3).

Over the last decade, molecular subtypes of BC based on whole genome expression profiles have been proposed (4-8), and recently, the “Bladder Cancer Molecular Taxonomy Group” has achieved a consensus for molecular classification of muscle-invasive BC (9). Although the names used for the subtypes differed between these studies—in analogy to breast cancer subtypes—they can be divided into two major groups, *i.e.*, basal and luminal, with potential differences in histological patterns and clinical behavior (7).

Basal BCs are enriched for basal type cytokeratins (CK) 5, 6, and 14, that have been reported to serve as markers of basal urothelial cells and stem/progenitor cells, whereas luminal BCs express markers of terminal differentiation, including CK20, GATA3, and uroplakins (7,8).

Pathologists have identified histological variants of urothelial carcinoma, which may be considered as the phenotypic expression of tumor heterogeneity, possibly reflecting the underlying diversity in molecular composition (10). More recently, integrative morpho-molecular approaches have suggested that immunohistochemical (IHC) markers, used individually or in panels, could help categorize muscle-invasive urothelial carcinomas of the bladder (MIUCB) into these two major phenotypes (11,12).

In this study, we used two combinations of IHC markers to distinguish subtypes, searching for their association with clinicopathological features, including the presence of histological variants and outcomes. In addition, we assessed tumor heterogeneity by comparing the subtype characterized in the MIUCB, with the subtype found in their matched lymph node metastasis.

## MATERIALS AND METHODS

### Study Cohort and Histological Evaluation

We retrospectively collected data from 300 consecutive patients who underwent cystectomy for primary urothelial carcinoma at two institutions. Two hundred thirteen patients were treated at ICESP, Hospital das Clínicas HCFMUSP, Faculdade de Medicina, Universidade de São Paulo, São Paulo, SP, Brazil, between January 1, 2009, and December 31, 2013, and 87 patients were treated at Hospital Alemão Oswaldo Cruz, São Paulo, SP, Brazil between January 1, 2005, and December 31, 2014. This study was approved by the Institutional Review Board of Faculdade de Medicina, Universidade de São Paulo (approval number 086/15; 03/18/2015) and by the Institutional Review Board of Hospital Alemão Oswaldo Cruz (approval number 1.633.980).

All hematoxylin and eosin slides were reviewed by one author (JNR) and 117 patients were excluded because of the following reasons: unavailability of paraffin blocks (23 cases), residual tumor size less than 1.0 cm (23 patients), stages pT0, pTa, and pT1 (29 patients), low-grade (11 patients), neoadjuvant chemotherapy (7 patients), partial cystectomy (4 patients), small cell carcinoma (4 patients), extensive squamous variant with a dubious urothelial carcinoma component (3 cases), and synchronous upper urinary tract carcinoma (1 patient). The final study cohort included 183 patients with high-grade MIUCB.

Data regarding sex, age, tumor size (≤3.0 cm *versus* >3.0 cm) (13), and focality were collected from the original pathology reports. Histological features such as lymphovascular invasion (LVI), perineural invasion, tumoral necrosis, associated carcinoma “*in situ*” (CIS), squamous, glandular, micropapillary, sarcomatoid, and plasmacytoid variants, peritumoral inflammation (PTI), intratumoral lymphocytes (ITL), pathological stage by AJCC/TNM 2010 (14), and lymph node metastasis (LNM), were evaluated through a thorough review of all slides containing panoramic sections. All samples were confirmed as high-grade urothelial carcinomas.

LVI was defined as the presence of tumor cells within or invading the endothelial-lined lymphatic space or muscle-lined blood vessels. Perineural invasion was defined as tumor invasion into the perineural sheath or endoneurium. Tumor necrosis was defined as coagulative necrosis within the tumor. CIS was defined by the presence of a flat lesion—in which the surface epithelium contains cells that are cytologically malignant—adjacent to the tumor, or in areas representative of other bladder walls. Any percentage of histological variants observed throughout the tumor was considered. Squamous, glandular, micropapillary, sarcomatoid, and plasmacytoid variants were characterized based on the 2004 WHO classification (2). PTI was assessed at the invasive front (defined as the deepest interface of carcinoma with stroma), distant from necrotic areas, or from inflammation secondary to previous surgical resection. Only lymphocytes were included in the assessment, the outcomes of which were categorized as absent/low and moderate/intense. ITL was also assessed in preserved tumors, distant from necrotic areas or from inflammation secondary to previous surgical resection, observed with a 40x microscope objective, and divided into the following three categories: absent, low/moderate (up to 2 lymphocytes/high-power field), and intense (more than 2 lymphocytes/high-power field) (15).

Follow-up data were available for the 120 patients from ICESP, and the outcomes of interest were overall survival (OS), cancer-specific survival (CSS), and recurrence-free survival (RFS). OS was defined as the interval between the date of surgery and death from any cause. CSS was defined as the interval between the date of surgery and death because of MIUCB. And RFS was defined as the interval between the date of surgery and the first documented clinical or radiological recurrence, or until the last follow-up.

### Tissue Microarray (TMA) Construction

Selected representative well-preserved areas of MIUCB in two different slides and their respective LNM, were chosen and marked during slide review. The donor paraffin blocks were punched in these areas of interest using a precision instrument (Manual Tissue Microarrayer, Beecher Instruments, Inc., Sun Prairie, WI, United States), and the tumor and metastatic tissue were transferred to a recipient block. In our strategy aimed at constructing TMAs generously representative of different components of primary and metastatic tumors, all 183 MIUCB were represented in five TMAs containing 1.0 mm cores, with 174 samples in quadruplicate cores, three samples in triplicate cores, and six samples in duplicate cores. All 76 LNM were represented in two TMAs, with 53 samples in duplicate cores, 13 samples in quadruplicate cores, 3 samples in triplicate cores, and 7 samples in only one core.

### Immunohistochemistry

CK20 and GATA3 were chosen to represent the luminal subtype, and CK5 was chosen to represent the basal subtype, as they are widely used in routine surgical pathology diagnosis and have previously been used for subtyping MIUCB (7,11).

IHC was performed on paraffin-embedded 3 µm sections from the TMA blocks, and the sections were deparaffinized and rehydrated. Antigen retrieval consisted of submerging the sections in 10 mM citrate buffer pH 6 (Merck, United States) and steam heating for 40 min. After washing with distilled water, endogenous peroxidase was blocked by incubating with 6% hydrogen peroxide in methanol at 20° to 25°C for 10 min, and this process was repeated three times. Universal protein blocking was performed by incubating with CAS-Block™ (Zymed, United States) at 37°C for 10 min. Antigen detection with the primary antibody was carried out by incubation at 37°C for 30 min, and then at 4°C for 18h. Signal amplification was achieved using the Novolink™ max polymer system (Newcastle, United Kingdom) with incubation at 37°C for 30 min. The immunoreactive signal was visualized by first incubating with chromogen 3-3′-diaminobenzidine (Sigma, USA) at 37°C for 5 min, followed by washing with distilled water, counterstaining with Harris’ hematoxylin by incubating for 1 min, dehydration in a progressive alcohol series, clearing in xylene, and mounting with Entellan™ (Merck, United States). Adequate positive and negative controls were selected for each primary antibody. Table 1 summarizes the IHC protocols and the respective antibodies used in this study.

### Immunohistochemistry Evaluation

The extent of CK5 and CK20 cytoplasmic/membrane expression and the extent of GATA3 nuclear expression were inspected visually under the microscope by a pathologist (JNR), and annotated for each TMA core using a semi-quantitative scale with 11 possible classes from 0 to 100% positive tumor cells (0%, 10%, 20%, 30%, 40%, 50%, 60%, 70%, 80%, 90%, and 100%). Friedman tests were used to compare the values obtained in the multiple cores from the same case, and as no significant difference was found between the cores, the highest value (hot spot) was used to represent the case.

A cut-off value of 20% tumor cells cytoplasmic/membrane (for CK5 and CK20) or nuclear (for GATA3) positivity was employed, as recommended by Dadhania et al. (11), and samples were considered negative if the expression of the target protein in IHC was less than or equal to 20% and positive if the expression of the target protein in IHC was greater than 20%.

**Figure 1:**
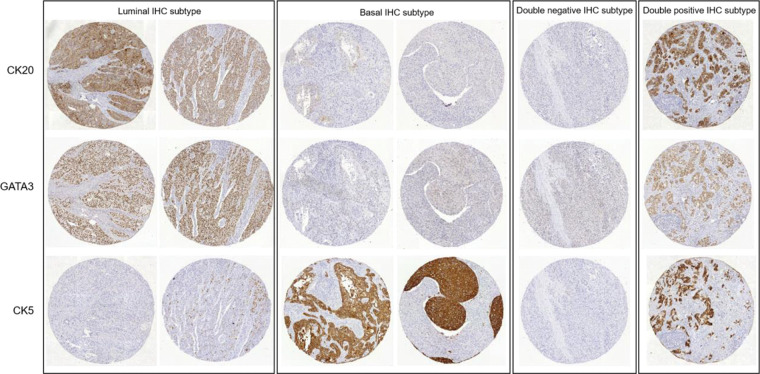
TMA cores stained with CK20, GATA3 and CK5 showing representative cases grouped as luminal, basal, double-negative and double-positive immunohistochemical subtypes.

As recommended by Dadhania et al. (11), patients who were CK5 positive and CK20 negative, or CK5 positive and GATA3 negative, were grouped in the basal subtype. Patients who were CK5 negative and CK20 positive, or CK5 negative and GATA3 positive, were grouped in the luminal subtype. Patients who were positive or negative for both marker sets were grouped as double-positive or double-negative subtypes, respectively. TMA cores stained with CK20, GATA3, and CK5 representative of the patients grouped into these four subtypes are shown in Figure 1.

Similarly, 73 out of 76 patients with LNM were classified using CK5 and CK20. It was not possible to classify three LNMs because of total loss of TMA tissue cores in the CK20 slide. All 76 LNMs were classified as CK5 and GATA3.

### Statistical Analysis

Statistical analyses were performed using R (version 3.5.1; R Foundation for Statistical Computing, Vienna, Austria). http://www.R-project.org/) and SPSS version 23.0 (SPSS Inc., United States). Pearson’s chi-square test or likelihood-ratio test was used to evaluate the association between categorical variables. The McNemar-Bowker test and Kappa coefficient were used to compare subtypes identified in tumors and LNM. Kaplan-Meier method was used to estimate survival functions, and differences were assessed using the Breslow test. A value of *p*<0.05 was considered significant.

## RESULTS

The clinicopathological features of the 183 high-grade MIUCB patients are summarized in Table 2. The patient ages ranged from 37 to 88 years (mean 66.3 years); the male-to-female ratio was 2.4:1. Tumor sizes were clearly reported in all except 2 cases, with tumor diameter ranging from 1.2 to 11.0 cm.

When the IHC subtypes were defined by markers CK5 and CK20, luminal IHC subtype was found to be significantly associated with micropapillary (*p*<0.001) and plasmacytoid (*p*=0.008) variants; low/moderate ITL (*p*=0.002) and less tumoral necrosis (*p*=0.025) when compared to the other groups. Luminal and double-positive IHC subtypes were associated with CIS (*p*=0.005). Any luminal IHC subtype case had glandular variants (*p*=0.029). In contrast, the basal IHC subtype was associated with the squamous variant (*p*<0.001), and none had multifocality (*p*=0.002) when compared to the other subtypes. Double-negative followed by basal IHC subtypes were associated with sarcomatoid variant (*p*<0.001). The double-negative IHC subtype was also associated with tumor size ≤3.0 cm (*p*=0.039) (Table 2).

When the IHC subtypes were defined by markers CK5 and GATA3, luminal IHC subtype was found to be significantly associated with micropapillary (*p*<0.001) and plasmacytoid (*p*=0.014) variants and less tumoral necrosis (*p*=0.005), when compared to the other groups. In contrast, the basal IHC subtype was significantly associated with female sex (*p*=0.014), squamous variant (*p*<0.001), moderate/intense PTI (*p*=0.008), and intense ITL (0.001), and none had multifocality (*p*=0.021). Basal—followed by double-negative IHC subtypes—were associated with tumor size >3.0 cm (*p*=0.028). The double-negative IHC subtype was associated with the sarcomatoid variant (*p*<0.001) when compared to the other groups (Table 3).

Lymphovascular and perineural invasions were detected in 148 (80.9%) and 123 (67.2%) cases, respectively. Regional lymph nodes were evaluated in 172 cases, of which 76 (44.2%) had LNM. The tumor stage was pT2 in 44 (24.0%), pT3 in 94 (51.4%), and pT4 in 45 (24.6%) cases. No significant association was observed between these four histopathological features and IHC subtypes using either pair of the markers.

The comparison of the IHC subtypes at the MIUCB *versus* their respective LNM showed no significant difference in the distribution between the four groups, based either on the combination of CK5/CK20 (*p*=0.440) (Table 4), or that of CK5/GATA3 (*p*=0.732) (Table 5). Moderate agreement was observed between the subtypes identified in MIUCB and LNM using both the combination of CK5/CK20 (kappa =0.58) (Table 4) and that of CK5/GATA3 (Kappa=0.51) (Table 5).

**Figure 2:**
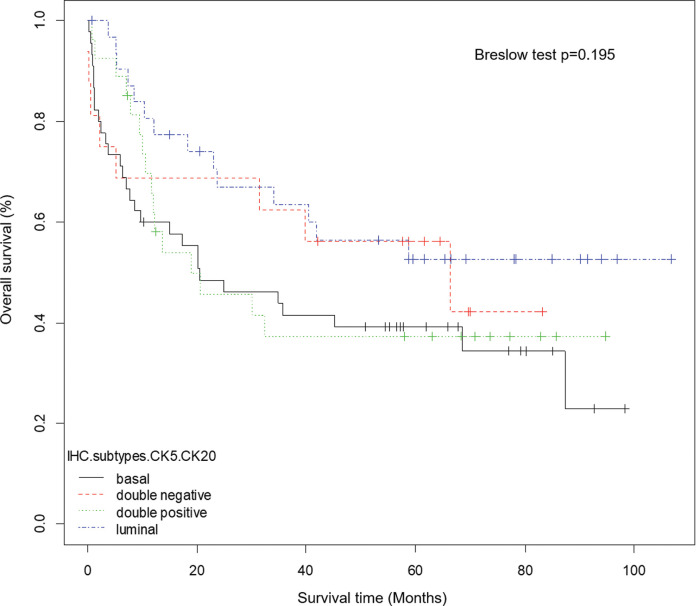
Overall survival of 120 patients treated with radical cystectomy for MIUCB. Kaplan-Meier curves showing no difference based on IHC subtypes using CK5 and CK20 (Breslow test *p*=0.195).

At a median follow-up of 30.7 months (0-106.8 months), 56 out of 120 (46.7%) patients from ICESP experienced disease recurrence after radical cystectomy, 43 (35.8%) died of MIUCB, and 67 (55.8%) died of any cause. Kaplan-Meier estimates were generated for OS (Figure 2), CSS, and RFS stratified by IHC subtypes using CK5 and CK20. Breslow tests showed no statistically significant difference between patients with basal, luminal, double-negative, or double-positive IHC subtypes (OS, *p*=0.195; CSS, *p*=0.318; RFS, *p*=0.42). Similarly, Kaplan-Meier estimates were generated for OS, CSS, and RFS stratified by IHC subtypes using CK5 and GATA3, and Breslow tests showed no statistically significant difference between patients with basal, luminal, double-negative, or double-positive IHC subtypes (OS, *p*=0.261; CSS, *p*=0.156; RFS, *p*=0.333).

## DISCUSSION

Comprehensive RNA expression profiling studies have identified at least five BC molecular subtypes, with basal and luminal divisions being the most fundamental (16). These two subtypes are believed to represent the opposite extremes in a spectrum of genetic alterations. Despite the different nomenclatures used by the many groups studying BC subtypes, there is a marked agreement on molecular alterations, suggesting common biological characteristics among tumors of the same subtype. Since systemic treatment for BC has been limited to cisplatin-based chemotherapy for several decades, with little progress in this period, these studies can lead to new and more individualized therapeutic options based on altered molecular pathways for each molecular subtype, with the aim of improving patient outcomes and quality of life (16).

In this context, similar to what is currently done for breast carcinomas, many authors (11,17-19) have proposed the use of IHC markers as surrogate markers for molecular classification of BC, since this can be easily done as part of daily clinical routines at low costs.

The two combinations of IHC markers used in this study associated micropapillary and plasmacytoid variants with the luminal IHC subtype, and squamous variant with the basal IHC subtype. These findings are consistent with those of previous studies (7,18,20).

Guo et al. (20) performed gene expression profiling and IHC in TMAs containing 43 micropapillary BC and demonstrated that this variant is almost exclusively luminal. In a recent study, Yang et al. (21) used IHC in whole tissue sections from 56 bladder urothelial carcinomas with micropapillary variant and showed that all of them expressed luminal markers (CK18, CK20, GATA3) and none were classified as the basal subtype.

Choi et al. (7), using gene expression profiling, showed that basal MIUCB in the discovery and validation cohorts with 73 and 57 cases, respectively, were significantly enriched with squamous features. They also associated the sarcomatoid variant with the basal subtype in the discovery cohort, although the small number of cases. It was quite similar to our findings using both combinations of markers, which also associated this variant with the double-negative IHC subtype.

The association of the plasmacytoid variant with the luminal IHC subtype was first suggested by Warrick et al. (18) in 2017, based on the IHC expression of CK14 (basal marker) and FOXA1 (luminal marker) in TMAs containing 309 BC, 35% of which presented histologic variants. Their data showed that plasmacytoid tumors tended to express high levels of FOXA1, suggesting luminal characteristics, but half of them also expressed high levels of CK14. They also demonstrated that the squamous variant tends to be basal (low FOXA1, high CK14), while micropapillary variant tends to be luminal (high FOXA1, low CK14).

Our finding of the association of the female sex with the basal IHC subtype using CK5/GATA3 is similar to the results of the study by Sjödahl et al. (5), which also found a greater proportion of female patients in the group characterized by high expression of basal keratins, squamous variant, and poor prognosis, suggesting that females are more likely to develop urothelial carcinomas with a keratinized/squamous phenotype associated with an adverse prognosis.

Robertson et al. (22) assessed 412 muscle-invasive BC using multiple analytical platforms, and demonstrated that the basal-squamous subtype showed the strongest immune expression signature, as well as the presence of lymphocytic infiltrates. Accordingly, we also found moderate/intense PTI and intense ITL associated with the basal IHC subtype obtained with the combination of CK5 and GATA3, suggesting that these tumors might be conductive to anti-PDL1 treatment. The study also observed strong expression of CIS signature genes in the basal-squamous subtype, suggesting that they develop from basal cells and CIS lesions. In contrast, we found that the histologically detected CIS was associated with the luminal and double-positive IHC subtypes, obtained with the combination of CK5 and CK20. This indicates that the method used for determining the presence of CIS (gene expression *versus* histology) might determine this difference.

Multifocality was not observed in any case subtyped as basal, using either pair of markers. Thomsen et al. (23) used exome sequencing to classify the samples from three non-invasive papillary urothelial carcinomas obtained from the same patient (multifocal tumor), as luminal subtype; however, the fourth tumor focus that was muscle-invasive showed a basal subtype, emphasizing the importance of performing multi-regional analysis for subtyping.

In our analysis of survival, there was no difference found between patients with basal, luminal, double-negative, or double-positive IHC subtypes, using either combination of markers. This is in contrast with the findings of other studies, that showed significantly shorter survival for the basal subtype (5-7,11).

An interesting finding from the present study was that there was only moderate agreement between the IHC subtypes identified in MIUCB and their respective LNM, using either combination of the markers, emphasizing that in the two comparisons, any case classified as luminal in the tumor was classified as basal in its LNM, and any case classified as basal in the tumor was classified as luminal in its LNM. Recently, Sjödahl et al. (24) assessed the concordance of molecular subtypes in 67 pairs of muscle-invasive BC and synchronous LNM, using IHC in TMAs and mRNA profiling for 57 BC and 28 matched LNM. These authors showed that discordant subtype classification between BC and LNM was not frequent (18%), but most (58%) involved the basal/squamous-like subtype. These discordant basal/squamous-like tumors showed either urothelial-like or genomically unstable, luminal-like phenotypes in the LNM, and the full section IHC in these tumors revealed intratumoral subtype heterogeneity in some of them. These findings raise the possibility of changing subtypes as a result of interactions with the microenvironment in the two anatomical sites, and also reinforce the concept of intratumoral heterogeneity also presented by Warrick et al. (25), showing different IHC subtypes in different areas from the same tumor presenting conventional urothelial carcinoma and histologic variants.

There are certain limitations to our study, such as its retrospective design and the fact that the follow-up evaluation was not available for all cases. Moreover, although all cases from our study primarily underwent cystectomy, we could not discriminate between patients who received adjuvant chemotherapy. With regard to immunohistochemistry, several other IHC markers have been shown to be associated with basal and luminal BC. Thus, we cannot exclude the possibility that IHC subclassification based on the use of different markers might yield different results. Furthermore, although we followed the most convincing description of cut-offs available in the literature, the results might also vary if other quantification methods and other cut-off values for IHC expression were applied.

The increasing knowledge and understanding of the genomic landscape, in addition to the molecular subtypes of MIUCB, will enable the development of new and precise biomarker-directed therapies. Therefore, a method to subtype the tumors that is applicable to routine clinical use is necessary (26).

In conclusion, beyond further stressing the need to describe each histological variant as a morphological evidence of tumor heterogeneity, this study demonstrates that using just three routine IHC markers, it is possible to stratify a significant number of MIUCBs into basal and luminal subtypes, which reveals differences in pathological features.

## AUTHOR CONTRIBUTIONS

Ravanini JN collected the data, reviewed all the histological samples, interpreted the results of immunohistochemical analyses, performed statistical analysis, and wrote the manuscript. Assato AK constructed the tissue microarrays and performed immunohistochemical analyses. Wakamatsu A performed immunohistochemical analyses and contributed to the manuscript drafting. Alves VAF reviewed histological samples, assisted in the interpretation of the results of immunohistochemical analyses, and participated in data interpretation, manuscript preparation, and critical revisions. All authors have approved the final version of the manuscript.

## Figures and Tables

**Table 1:** Summary of immunohistochemical procedures.

Antibody	Clone	Retrieval method^1^	Dilution	Staining pattern
CK5	XM26 (Novocastra)	Standard	1:800	Cytoplasm/membrane
CK20	Ks20.8 (Biocare Medical)	Standard	1:100	Cytoplasm/membrane
GATA3	L50-823 (Zeta Corp)	Standard	1:100	Nucleus

1 Antigen retrieval was performed by steam heating for 40 min in 10 mmol/L citrate buffer, pH 6.

**Table 2:** Associations between IHC MIUCB subtypes and clinicopathological features obtained using CK5 and CK20.

Clinicopathological features	No (%)	CK5/CK20				
		basal	luminal	double negative	double positive	*p* value
All cases	183 (100.0)	66 (36.0)	58 (31.7)	21 (11.5)	38 (20.8)	
Sex						
male	130 (71.0)	43 (65.2)	44 (75.9)	16 (76.2)	27 (71.1)	0.564^a^
female	53 (29.0)	23 (34.8)	14 (24.1)	5 (23.8)	11 (28.9)	
Tumor size*						
≤3.0 cm	64 (35.4)	16 (24.2)	22 (39.3)	12 (57.1)	14 (36.8)	**0.039^a^**
>3.0 cm	117 (64.6)	50 (75.8)	34 (60.7)	9 (42.9)	24 (63.2)	
Multifocality						
absent	169 (92.3)	66 (100.0)	50 (86.2)	18 (85.7)	35 (92.1)	**0.002^b^**
present	14 (7.7)	0 (0.0)	8 (13.8)	3 (14.3)	3 (7.9)	
Tumoral necrosis						
absent	64 (35.0)	16 (24.2)	29 (50.0)	7 (33.3)	12 (31.6)	**0.025^a^**
present	119 (65.0)	50 (75.8)	29 (50.0)	14 (66.7)	26 (68.4)	
CIS						
absent	109 (59.6)	48 (72.7)	25 (43.1)	15 (71.4)	21 (55.3)	**0.005^a^**
present	74 (40.4)	18 (27.3)	33 (56.9)	6 (28.6)	17 (44.7)	
Squamous variant						
absent	134 (73.2)	35 (53.0)	53 (91.4)	18 (85.7)	28 (73.7)	**<0.001^a^**
present	49 (26.8)	31 (47.0)	5 (8.6)	3 (14.3)	10 (26.3)	
Glandular variant						
absent	172 (94.0)	61 (92.4)	58 (100.0)	19 (90.5)	34 (89.5)	**0.029^b^**
present	11 (6.0)	5 (7.6)	0 (0.0)	2 (9.5)	4 (10.5)	
Micropapillary variant						
absent	144 (78.7)	61 (92.4)	35 (60.3)	17 (81.0)	31 (81.6)	**<0.001^a^**
present	39 (21.3)	5 (7.6)	23 (39.7)	4 (19.0)	7 (18.4)	
Sarcomatoid variant						
absent	166 (90.7)	56 (84.8)	58 (100.0)	16 (76.2)	36 (94.7)	**<0.001^b^**
present	17 (9.3)	10 (15.2)	0 (0.0)	5 (23.8)	2 (5.3)	
Plasmacytoid variant						
absent	174 (95.1)	66 (100.0)	51 (87.9)	20 (95.2)	37 (97.4)	**0.008^b^**
present	9 (4.9)	0 (0.0)	7 (12.1)	1 (4.8)	1 (2.6)	
PTI						
absent/low	91 (49.7)	26 (39.4)	28 (48.3)	12 (57.1)	25 (65.8)	**0.064^a^**
moderate/intense	92 (50.3)	40 (60.6)	30 (51.7)	9 (42.9)	13 (34.2)	
ITL						
absent	33 (18.0)	13 (19.7)	8 (13.8)	6 (28.6)	6 (15.8)	
low/moderate	131 (71.6)	41 (62.1)	50 (86.2)	12 (57.1)	28 (73.7)	**0.002^b^**
intense	19 (10.4)	12 (18.2)	0 (0.0)	3 (14.3)	4 (10.5)	

^a^ *p* value obtained using Pearson’s chi-square test; ^b^ *p* value obtained using likelihood-ratio test. *Tumor size not available in 2 cases. Boldface values are those that are significant (*p*<0.05). Abbreviations: IHC, immunohistochemical; MIUCB, muscle-invasive urothelial carcinomas of the bladder; CIS, carcinoma “*in situ*;” PTI, peritumoral inflammation; ITL, intratumoral lymphocytes.

**Table 3:** Associations between IHC MIUCB subtypes and clinicopathological features obtained using CK5 and GATA3.

Clinicopathological features	No (%)	CK5/GATA3				
		basal	luminal	double negative	double positive	*p*
All cases	183 (100.0)	25 (13.7)	73 (39.9)	6 (3.3)	79 (43.1)	
Sex						
male	130 (71.0)	11 (44.0)	56 (76.7)	4 (66.7)	59 (74.7)	**0.014^a^**
female	53 (29.0)	14 (56.0)	17 (23.3)	2 (33.3)	20 (25.3)	
Tumor size*						
≤3.0 cm	64 (35.4)	4 (16.0)	33 (46.5)	1 (16.7)	26 (32.9)	**0.028^b^**
>3.0 cm	117 (64.6)	21 (84.0)	38 (53.5)	5 (83.3)	53 (67.1)	
Multifocality						
absent	169 (92.3)	25 (100.0)	63 (86.3)	5 (83.3)	76 (96.2)	**0.021^b^**
present	14 (7.7)	0 (0.0)	10 (13.7)	1 (16.7)	3 (3.8)	
Tumoral necrosis						
absent	64 (35.0)	3 (12.0)	35 (47.9)	1 (16.7)	25 (31.6)	**0.005^a^**
present	119 (65.0)	22 (88.0)	38 (52.1)	5 (83.3)	54 (68.4)	
CIS						
absent	109 (59.6)	18 (72.0)	36 (49.3)	4 (66.7)	51 (64.6)	0.125^a^
present	74 (40.4)	7 (28.0)	37 (50.7)	2 (33.3)	28 (35.4)	
Squamous variant						
absent	134 (73.2)	9 (36.0)	66 (90.4)	5 (83.3)	54 (68.4)	**<0.001^a^**
present	49 (26.8)	16 (64.0)	7 (9.6)	1 (16.7)	25 (31.6)	
Glandular variant						
absent	172 (94.0)	24 (96.0)	71 (97.3)	6 (100.0)	71 (89.9)	0.199^b^
present	11 (6.0)	1 (4.0)	2 (2.7)	0 (0.0)	8 (10.1)	
Micropapillary variant						
absent	144 (78.7)	25 (100.0)	46 (63.0)	6 (100.0)	67 (84.8)	**<0.001^a^**
present	39 (21.3)	0 (0.0)	27 (37.0)	0 (0.0)	12 (15.2)	
Sarcomatoid variant						
absent	166 (90.7)	19 (76.0)	71 (97.3)	3 (50.0)	73 (92.4)	**<0.001^b^**
present	17 (9.3)	6 (24.0)	2 (2.7)	3 (50.0)	6 (7.6)	
Plasmacytoid variant						
absent	174 (95.1)	25 (100.0)	65 (89.0)	6 (100.0)	78 (98.7)	**0.014^b^**
present	9 (4.9)	0 (0.0)	8 (11.0)	0 (0.0)	1 (1.3)	
PTI						
absent/low	91 (49.7)	5 (20.0)	37 (50.7)	3 (50.0)	46 (58.2)	**0.008^b^**
moderate/intense	92 (50.3)	20 (80.0)	36 (49.3)	3 (50.0)	33 (41.8)	
ITL						
absent	33 (18.0)	1 (4.0)	13 (17.8)	1 (16.7)	18 (2.8)	**0.001^b^**
low/moderate	131 (71.6)	15 (60.0)	58 (79.5)	4 (66.7)	54 (68.4)	
intense	19 (10.4)	9 (36.0)	2 (2.7)	1 (16.7)	7 (8.9)	

^a^ *p* value obtained using Pearson’s chi-square test; ^b^ *p* value obtained using likelihood-ratio test. *Tumor size not available in 2 cases. Boldface values are those that are significant (*p*<0.05). Abbreviations: IHC, immunohistochemical; MIUCB, muscle-invasive urothelial carcinomas of the bladder; CIS, carcinoma “*in situ*;” PTI, peritumoral inflammation; ITL, intratumoral lymphocytes.

**Table 4:** Comparison of the IHC subtypes obtained in the MIUCB and in the respective LNM using CK5 and CK20.

	LNM IHC subtype CK5/CK20 n (%)				
	basal	luminal	double negative	double positive	Total
MIUCB IHC subtype CK5/CK20					
basal	16 (21.9)	0 (0.0)	2 (2.7)	4 (5.5)	22 (30.1)
luminal	0 (0.0)	22 (30.1)	2 (2.7)	5 (6.8)	29 (39.7)
double negative	0 (0.0)	2 (2.7)	5 (6.8)	1 (1.4)	8 (11.0)
double positive	1 (1.4)	5 (6.8)	0 (0.0)	8 (11.0)	14 (19.2)
Total	17 (23.3)	29 (39.7)	9 (12.3)	18 (24.7)	73 (100.0)

McNemar-Bowker test: *p* value=0.440; Kappa coefficient=0.58.

Abbreviations: IHC, immunohistochemical; MIUCB, muscle-invasive urothelial carcinomas of the bladder; LNM, lymph node metastasis.

**Table 5:** Comparison of the IHC subtypes obtained in the MIUCB and in the respective LNM using CK5 and GATA3.

	LNM IHC subtype CK5/GATA3 n (%)				
	basal	luminal	double negative	double positive	Total
MIUCB IHC subtype CK5/GATA3					
basal	4 (5.3)	0 (0.0)	0 (0.0)	4 (5.3)	8 (10.5)
luminal	0 (0.0)	28 (36.8)	2 (2.6)	6 (7.9)	36 (47.4)
double negative	0 (0.0)	1 (1.3)	2 (2.6)	0 (0.0)	3 (3.9)
double positive	2 (2.6)	8 (10.5)	0 (0.0)	19 (25.0)	29 (38.2)
Total	6 (7.9)	37 (48.7)	4 (5.3)	29 (38.2)	76 (100.0)

McNemar-Bowker test: *p* value=0.732; Kappa coefficient=0.51.

Abbreviations: IHC, immunohistochemical; MIUCB, muscle-invasive urothelial carcinomas of the bladder; LNM, lymph node metastasis.
